# Foreign

**Published:** 1840-12-05

**Authors:** 


### FOREIGN.

*Practical Observations on the Diseases of Peru, described as they occur on the Coast and in the Sierra.* By ARCHIBALD SMITH, M. D.

I.—DISEASES OF THE COAST CONTINUED.

*Diarrhœa.*—Among the diseases which daily require the anxious attention of the Lima practitioner, are diarrhœa and dysentery. And these diseases are at no season more likely to occur in an untoward form, than during the winter months, after the mists and cloudy weather have fairly set in.

When the fine weather of summer is prolonged throughout a considerable portion of autumn, the inhabitants of the coast still continue the light dresses, as well as evening walks and promenades to which they are used in summer; yet, from sunset to sunrise, they no longer breathe the warm breezes of the preceding months, but a cool, damp, and penetrating air.

During the hot sultry months the Limenians very generally indulge in the custom of resorting, especially early in the morning and at night, to the Palace or Cathedral Square, to refresh themselves with cooling beverages, of which a great variety is sold there. But when this custom, which is as safe and agreeable as it is cheering and sociable in the summer time, is continued after the season has changed, and when the night and morning air has become cool and damp, its effects are often injurious. Diarrhœa is one of the commonest disorders which occurs under these circumstances. And as conducing to this disease, when it prevails among the fair sex, I must not omit to advert to their passion for having their feet look exceedingly neat, by wearing the finest shoes and stockings. For these are very unsuitable for walking the streets of Lima with in time of *Garua*, or hazy and drizzly weather.

During winter, especially in the months of July and August, many persons are affected with vomiting of pure bile, and others with frequent liquid stools of hot and acrid quality, or with bilious diarrhœa, combined with more or less nausea and vomiting.

At this season, too, micturition is sometimes troublesome and frequent, more particularly with those travellers who have just arrived from the warm and dry inland valleys. They are so affected from the sudden transition of climate, and just in proportion as the transpiration from the surface of the body is cheeked. These ailments of internal congestion with augmented secretion, which may be traced to the external agency of cold and humidity as a general cause, are daily removed, because the balance of the circulation is restored, in a great majority of instances, by such means as open the skin, and keep up diaphoresis. But I may remark, that giving iced drinks, with a view of restraining the excessive secretion of bile, in the cases of bilious derangement and disturbance of the alimentary canal, which so often occur in the wet season, is not safe as a general practice, especially in persons of weakly constitutions, in whom a healthy reaction may not be readily induced. Cooling beverages and ice, indeed, are highly approved and recommended by daily experience, in the bilious disorders of the warm and dry season; but it should be remembered that then the nervous and vascular systems are too much stimulated by atmospherical heat, and the dermal capillaries are so particularly active, that iced drinks are not merely borne with safety, but freely used with great relish and comfort.

The exciting causes of diarrhœa are so numerous, that, even without any special regard to the season of the year or state of the atmosphere, the disorder may be daily traced to a great variety of other sources. Frequent liquid alvine discharges are often occasioned by the irritation or inflammation induced in the surface of the mucous membrane of the intestines, by fruit of various sorts, and in different stages of maturity; different articles of food, which are often voided in a half-digested state; and I may also mention the presence of worms in the bowels. Some chronic and very obstinate forms of diarrhœa are sustained by an ulcerated and thickened condition of the mucous membrane of the colon and rectum. I have seen severe cases of this character, in which relaxation of the *sphincter ani*, accompanied with a protrusiom of the rectum, took place, as often as there was occasion to go to stool.

As diarrhœa is one of the most common diseases on the coast, and, at the same time, one of no small danger when neglected or mismanaged, it may be convenient to detail the appearances under which, in cases of this nature, the alvine discharges usually present themselves. In this manner some idea may be formed of the nature and seat of the morbid causes which induce the various modifications which I shall endeavour to point out.

I specify, 1. *Diarrhœa distinguished by an increased mucous discharge.*—This is a common modification of the disorder, which indidates a catarrhal affection of the intestinal mucous membrane. It is rarely attended with any serious disturbance of the circulation or general system. The tongue is whitish, and often clammy; the pulse not much affected. The attack comes on with some chilliness or feeling of cold, often referred to exposure to night air. Those who are thus indisposed rarely require the help of the physician; for such slight disorders of the bowels are commonly removed by general warmth, rest, pediluvia, farinaceous diet, and diluents.

2. *Diarrhœa marked by a dry skin and thin serous stools.*—In this form of diarrhœa, the tongue is furred; the pulse considerably quicker than usual; appetite impaired; skin dry; the stools frequent, dark, fetid, and serous. I have observed cases of this kind occur in the misty or wet season, when it was found difficult or impossible to restore the skin to its naturally soft and perspirable state.

Absorbent medicines, aperients, and warm-baths, mitigate the symptoms of this disease; while, on the other hand, opiates and astringents are apt to cause tension of the abdomen, and aggravate the complaint. And these remedies are found to prove injurious in proportion as they restrain the intestinal secretion, which may be considered as vicarious of that of the skin. When medicines fail to correct this affection in Lima during winter, a visit of a few weeks to Chorillos should be recommended; for, in this favoured locality, the climate during the wet season is so benign, that it naturally tends to restore the proper balance of the circulation, and will often render the cutaneous vessels and excretories easily acted upon, by the application of those very remedies which, in Lima, might be long and sedulously used without success.

3. *Diarrhœa characterized by deficient biliary secretion.*—In strongly marked instances of this kind of diarrhœa, the most striking symptoms are, white evacuations, which are frequent and troublesome in course of the night; a dry skin; loss of appetite; depression of spirits; and, in protracted cases, general wasting of the flesh. Disorders of this character are more frequently met with in the chronic form. In every instance which came under my notice, the remedies used, with perfect success, were common aperients, and the immersion of the hands or feet for twenty minutes, or half an hour, morning and evening, in the nitro-muriatic acid bath.

4. *Diarrhœa characterized by an augmented biliary secretion.*—This is the modification of diarrhœa with which, above all others, the people of Lima are familiar. We have already seen that it is prevalent during the winter months; and in spring, when the sun shines forcibly in the middle of the day, one may complain of feeling chilly, and then some uneasiness is felt in the bowels; the tongue is foul; anorexia, perhaps nausea or bilious vomiting, take place; and slight headach, with some alteration in the pulse, follow. A call to stool comes in the train of the above symptoms, and relief is obtained from much griping by voiding several stools of a dark, fetid, and feculent appearance, mixed with a thin, yellow, frothy, and bilious discharge.

In the warm and dry season, we find the patient who complains of disordered bowels, which he attributes very often to immersion in the cold bath, with a white tongue, especially in the morning, affected with either drowsiness, general languor, and a distaste for food; and in the course of the day, some griping is felt, and soon followed by two or three loose stools. These evacuations are sometimes of a rhubarb colour; more generally of the colour of yolk of egg; often frothy, and voided with a sensation of scalding heat.

Cases like either of the above are usually treated very successfully by farinaceous diet, starch enemata, and as common drink, linseed and mallow water, sweetened with syrup of gum-arabic, or, two or three times in course of the day, a glassful is taken of an infusion made of tamarinds, linseed, roses, and mallows, in common water sweetened with syrup of roses. Perhaps the state of the bowels may require a purgative, in which case manna with rhubarb, made into a beverage with sugar and water, usually called *angelica*, is found to answer very well in most instances; for in these cases the remedial means are generally simple.

Disorders of the liver being very prevalent, hepatic patients are sometimes seen in Lima, who hardly ever perspire in summer or winter. They suffer habitually from a painful sensation of heat in the region of the liver, extending to the shoulder, or interscapular region. The tongue is furred, of a dirty white appearance. They endure but very little covering by day or by night; and they say that even their ordinary clothing is troublesome to them, by increasing the heat, or "ardor," which they always experience in the affected side, as well as between the shoulders. And though it is evident that, in such examples, the liver is seriously in fault, yet we may not be able to detect any palpable alteration in the structure of this organ, or any induration, unusual sensibility, or tumefaction, in any part of the abdomen.

Chronic ailments of this nature are alleviated by warm baths in the wet, and cold baths in the dry season. But persons who are affected in this way are obliged to observe a careful regimen, and are peculiarly subject to attacks of diarrhœa. At one time the motions are sanguineous, with or without a considerable portion of mucous matter; at other times, the evacuations are not sanguineous, but as yellow as broom blossoms, or as green as the clover leaf.

I have witnessed some cases in which the motions were copious, and quite of a bilious appearance, accompanied with dull pain at liver, a white and furred tongue, but without much of a febrile pulse.

Cases of this character, which are obviously sustained by great disorder in the functions or structure of the liver, are sometimes benefited by local bleeding and blistering, with bland diet, and laxative aperient remedies; or they may require the application of mercurials, aided by the warm bath; and, on many occasions, entire change of climate is required. In most instances, however, where we meet with a diarrhœa of long standing and hepatic origin, bleeding may be very well dispensed with, as the practitioner, on a review of all the circumstances, will be able to judge, and mercurial inunction may be safely and advantageously resorted to at once. And I may observe, that from the external application of mercury in the manner alluded to, aided by minute doses of calomel, I have witnessed the best results, in some cases of a chronic muco-sanguineous diarrhœa, when connected with the presence of a great many moveable tumours about the size of walnuts, situated within the abdomen. Both the tumours and diarrhœa disappeared as the salivary organs were slightly affected by the action of the mercury.

The worst forms of diarrhœa met with in Lima cannot be enumerated under any one of the general heads above specified ; for they are usually of a very mixed character. It often happens, that for some days the stools are entirely of a bilious aspect, and attended with very little alteration or acceleration in the pulse; but as these cases grow inveterate, they may pass into dysentery. And in other cases terminating in dysentery, the evacuations, for several days, or even weeks, consist of acrid matter of various appearance, or vitiated secretions of many colours, and more or less feculent quality; or, perhaps, yellow when voided, but soon becoming dark-looking on exposure to the air.

In those examples of diarrhœa, when the evacuations are of a mixed character, of a bilious and yet somewhat slimy and ropy appearance; of a greenish colour, and as many as seven or eight daily, the natives take alarm lest the disorder should end in real dysentery. The women more particularly are most observant of these signs of approaching danger; for from their childhood they are taught to watch, when in any degree indisposed, the appearance of the egesta, from which they are accustomed to infer what it may be safe for them to eat. One of these women, who is accustomed, when in health, to eat of every thing that she fancies, or comes in her way, is no sooner affected as I have just described, than, first, she starves herself, and then sends for the physician. She tells him, probably, that her illness has been coming on gradually for some days before; that since its commencement, she has carefully denied herself every kind of food, except a little "alaguita de mais blanco," or gruel of white maize meal, alternated with the standard drink on such occasions, an infusion of roses and linseed, slightly sweetened with the azucar rosada, or rose sugar. So much apprehension and caution on the part of the experienced natives, show better than the most elaborate discourse could do, the acknowledged common tendency of such ailments.

Mental affections, and disturbed states of mind, have often a remarkable share in producing various degrees of deranged digestion.

In some instances we find that depressing passions of the mind disorder the functions of the liver, debilitate the stomach, give rise to indigestion, and hence induce diarrhœa ; and that at other times, the exciting passions, as anger, cause very great disturbance in the bowels. Thus it is curious to observe how frequently bakers are subject to disorders of the bowels in Lima.

This is a class of men whose temper is incessantly tried, and often fretted in course of their daily avocations. The bake-houses are used as a kind of penitentiaries, or places of temporary penance and chastisement, wherein delinquent slaves are employed at hard labour. With these the bakers have to contend in the character of task-masters; and hence the conflict of opposing interests and passions, which renders the slave sullen, and keeps the overseer in a state of constant disquiet. Nor is it the baker alone who exhibits the effects of the mind over the body ; for persons of every class and occupation on the coast, whose dispositions happen to be quick and choleric, are peculiarly prone to diarrhœa. It is therefore a very general custom with such people to endeavour to counteract the known tendency of their irascible emotions, in altering the condition of the intestinal secretions, increasing the biliary secretion, and disturbing the bowels, by taking as preventives ices, and iced pine-juice in particular; by means of which, in hot weather at least, they are really refreshed, and relieved for a time. In ordinary examples, where diarrhœa is habitually induced from certain disagreeable states of mind, other exciting causes may also have a share in producing the same effects, as errors in diet, or too much bodily exercise, and frequent exposure to the sun. In bakers in particular, whose duties at certain hours consign them to the annoyances within doors, and, at others, oblige them to ride about a great deal in the sun, I have observed that they were often relieved of very severe attacks by their own popular remedies, viz., tepid, mallow, and aperient drinks, or whey with manna and tamarinds, and other emollient and soothing means in current use, as warm baths, cataplasms, unguents, and enema.

But when the diarrhœa continues in spite of these or other means, timeously tried, and is known to be kept up by the operation of moral causes preying on the mind, then we may expect to observe pyrexia come on, with more or less tenesmus; and, perhaps, the belly may at some point feel tender to the touch. Now the evacuations, whatever may have been their original character or appearance, begin to show some traces of blood, become less free and copious, but oftener repeated, and are, in this state, what the natives denominate "Picando in Bicho," that is, merging into an established dysentery.—*Edinburgh Med. and Surg. Jour.*

---

### M. GENDRIN ON UTERINE HÆMORRHAGE.

*Utero-Placental Hæmorrhage.*—This is the term used by our author to designate that uterine flux which occurs or may occur during the period of childbearing and childbirth. The whole subject is carefully considered, and some points brought out in a more salient position than is usually met with in books; still the vices of composition, apparent in the preceding chapters, are not omitted here. There is the same prolixity of detail and a similar intricacy of method, the former exhausting all patience, the latter destroying all simplicity and clearness. We shall cull the facts for the reader just as they occur, page by page, utterly despairing of bringing those which would serve mutually to illustrate each other into one point of view.

According to M. Gendrin, the physiological conditions of the uterus during gestation are favourable to flooding, inasmuch as there is not only a natural congestion but also a natural hæmorrhage in the interchange of the molecules of blood between the maternal and fœtal systems. Hence the frequency and the importance of the accident. The symptoms and the results on the frame are always of the same kind in every species of "utero-placental" discharge; but there are great differences as to the mode of manifestation and the succession of the phenomena in each—varieties which hinge chiefly on the fact as to the connexion between the ovum and the uterus. This connexion may either be normal or abnormal; the former when the placenta is attached to any portion of the uterine walls but the cervix, the latter when the placenta is implanted on the cervix.

A. Hæmorrhage with Normal Implantation of the Placenta.—This occurs most frequently before the twenty-fourth week of gestation, and generally coincides with the epoch at which the woman would have menstruated had she not been pregnant. This last remark of M. Gendrin is fully borne out in our own experience, and is strongly recommended as a basis of action. The precursory symptoms of discharge are those of uterine "hyperæmia," heat, fulness, and spasm of the womb, and sympothetic irritation of the bladder, kidneys, and rectum. The development of the muscular fibre of the uterus lays the foundation of spasm of that organ, and deceives the woman into a belief that the motion felt internally is attributable to the movements of the child, which, as M. Gendrin justly observes, is from its gelatinous state incapable of such action. These spasms soon become painful, and are accompanied by erratic flushes of heat and cold. After a few days the flux of blood is ushered in by weight of the loins and small of the back, feebleness, and fever. In severer forms there is great inequality of the circulation and even syncope; and usually in advanced pregnancy inordinate movement of the child. The blood may appear either externally or be concealed within the womb. This is a most important distinction as to practice.

1. *In external or open hæmorrhage* the blood may flow continuously or in interrupted jets, accompanied by grinding pain in the hypogaster and loins. With these pains an early ovum is rapidly expelled; but a longer period is required in a more advanced state of pregnancy. To this general description we would add a practical point for the junior practitioner: if there be flooding alone, or pain alternating with ease alone, abortion does not necessarily take place; but if there be both discharge and intermittent pain, the chances are that the ovum will be expelled. It is always to be wished that the whole ovum should be expelled at once in early gestation. A small portion of placenta or membrane left in the uterus will excite a flooding which, even at the third month, we have in more instances than one known as deeply affecting the actual and the future health of the sufferer. If the hæmorrhage does not cause expulsion of the ovum, it may still determine its death; a knowledge of which fact we can acquire with greater certainty in proportion to the advanced state of gestation. At all periods, however, there are symptoms the presence of which lead to the inference of suspended gestation; these are—1, the disappearance of those symptoms which had hitherto accompanied pregnancy, such as sickness, &c.; 2, cessation of development in the breasts and abdomen; 3, irritative presence of fever, marked by a feeble, hurried, unequal pulse, sunken features, tarnished skin, disordered hepatic function, and diarrhœa; 4, the uterus is flabby, the fœtus motionless, and not easily moved by the impulse of "ballottement." These cases are very troblesome, from the perpetual anxieties they cause both to patient and practitioner. They rarely go to the full time, yet occasionally they do. The labour is not, however, more liable to hæmorrhage, although the puerperal state is to its worst fever, than where the abortion takes place at once. The worst examples, however, in our own experience occur in twin cases, where one of the children has been blighted some weeks previous to birth.

2. *Latent or internal hæmorrhage* during gestation is much rarer than external or open flooding. The author speaks of its occurring frequently in the early months of pregnancy; and the general tenor of his context would lead us to believe that it then was similar in its march and effects to internal hæmorrhage in the later months of childbearing. Such certainly is not the fact. The uterus before the sixth month is rigid, indistensible, and incapable of holding so much blood as to endanger the life of the patient; and very rarely or never, but in the extremely feeble constitution, does the latent hæmorrhage cause the specific danger of an internal and concealed flooding. In a more limited sense, however, we agree with M. Gendrin when he classes under latent hæmorrhage those effusions of blood which take place in the placenta or the uterine membranes, giving rise to subsequent abortion and the death of the fœtus. These are similar to those effusions into the lungs of adults known as pulmonary apoplexy, and affect the immature organism by means analogous to the process of destruction in the mature one, viz., in speedily destroying life by impeding the vital changes of the blood.

The symptoms of latent utero-placental hæmorrhage shall be given in the author's own words:

"The primary symptoms of latent hæmorrhage are precisely the same as those characterising the open flux; nevertheless, the lumbar pains are severer and seem to threaten the expulsion of the ovum. There is at the same time a sense of general discomfort, feebleness, cold sweats bedewing the temples, chilliness, and such a tendency to fainting that the patient often is thrown into a continuous state of 'demi-syncope,' in the midst of which, nausea or vomiting produces complete prostration. These symptoms are variable as to duration, but they rarely last longer than four or five hours, and still more seldom do they flow in one uninterrupted course of increasing intensity, but are broken by intervals of remission, during which the patient suffers only from debility and general discomfort. The face, however becomes paler and the pulse more feeble." (p, 172.)

The symptoms are often less urgent, and recur at intervals of six or eight hours, each paroxysm being preceded by a repetition of those lumbar pains, those tremors, &c., which formed the percursory signs of the first attack. If the blood extravasated into the womb be in a certain quantity, it may become sensible to the external touch. Levret, J. Hopff, Leroux of Dijon, Baudelocque, have severally felt an accessory tumour jutting out the uterine walls.

M. Gendrin notices the union of sensible and latent hæmorrhage, under the designation of "demi-latent," for the purpose of stating that the expulsion of the ovum is generally in such cases certain, and accompanied, during the whole process, with a discharge, entailing on the constitution the effects of an abundant flooding.

If, in spite of these discharges, pregnancy persists, the fœtus may remain in utero, as a foreign body, causing a valetudinary state of health, characterized by symptoms of incessant impending abortion: or there may be a gradual increase of the volume of the uterus, dependent on a chronic latent hæmorrhage, when all the rational signs of pregnancy have ceased with the blighting of the fœtus. In these instances, the limbs become anasarcous, the digestion difficult, the body enfeebled and meager, the bowels relaxed, the urine scanty, and, finally, hectic fever sets in with a tendency to fainting. This state of things may endure up to the full period of gestation, bringing the patient to the brink of the grave by their exhausting influences. The placenta is found to be, in every instance, larger than in natural labours, its increased bulk being dependent on blood which has become imbedded and entangled in its cellular texture; and with this mass a large quantity of grumous fluid is usually expelled. These labours are less subject to subsequent hæmorrhage than any others, a fact quite accordant with our own experience, and one explicable by that *chronic disruption* of the utero-placental bonds, which affords sufficient time for the vessels of the womb to retract and become obliterated.

There are cases of a fatal termination to latent hæmorrhage. Some of these M. Gendrin has narrated, and they are well worth attention; indeed the whole subject of latent, or, as we term it, internal hæmorrhage, is not sufficiently known in all its bearings in this country, although it is by far the most difficult and appalling of the more serious complications of gestation.

"A woman who had remained in the clinic of a private teacher two days was brought," says M. Gendrin, "to our hospital, for pains which seemed to announce labour. She was in her seventh month of pregnancy, and hitherto had not experienced any untoward symptom. The pains were slight, and occurring at distant intervals. In order to hasten birth, ergot was given, but without effect. On the second day, all the symptoms of loss of blood being present, she was brought from the private clinic to the hospital. The abdomen was soft, the uterus reached to the epigastrium, its os was closed, and neither the beat of the fœtal heart nor the placental murmur could be heard with the stethoscope. The patient expired in two hours. The ovum was found enveloped in a mass of blood, partially coagulated. The placenta had been torn from its uterine connections by coagula, which covered two-thirds of the upper part of the chorion. A zone, of about three inches in breadth, of adherent chorion, formed a dam between the clots and the uterine orifice. The placenta was infiltrated with blood. The liquor amnii was slightly red. The fœtus appeared six months old, and exhibited no trace of decomposition. The umbilical vessels, veins, and arteries, were distended with coagulated blood." p. 180.

In another instance Albinus found the central portion of the placenta pouched out with blood, which had nevertheless not destroyed its marginal uterine adhesion. We are acquainted with an example in which the patient was seized at the seventh month of pregnancy with constant faintings and all the symptoms of internal hæmorrhage. Delivery was forcibly produced, and the amnion was found filled with coagula. In our own experience these dangerous forms of latent hæmorrhage, prior to the ninth month, are rare. They are seldom wholly internal in early gestation; seldom, therefore, do they put the practitioner in doubt as to the course of action requisite to save his patient. Where the flooding is concealed, it is of the greatest moment to arrive speedily at a knowledge of the fact. But how is this to be done amid the tumult of anxieties which, in these disastrous examples, spring up in the lying-in chambers to perplex the practitioner? We have been accustomed to watch with extreme care the state of the respiration; for unless the gush be at once immense and sudden (a rare case) the lungs exhibit the effect of flooding, before the brain gives way or the pulse alters much. The long-drawn frequent sigh, the repeated indraughts of air which take place in incessant yawning, prove that the balance between the lungs and the heart is disturbed, and this alone has often induced us to linger at the bedside to ward off a hæmorrhage which we knew by these signs had already begun. And let it be remembered that these early moments are those on which health and life hang. The uterus is yet full of contractile power, and readily reacts now; while a few enlarged gushes, unrestrained, exhausting irritability, render hæmorrhage a cause of additional flooding.

Among the earliest symptoms, then, of loss of blood is some unusual state of respiration; and in an interval, (whose length is modified by the velocity of the flux, its quantity, and by the previous constitutional state of the patient,) the pulse begins to falter, and the brain to become dizzy with faintness.

3. *Flooding during labour.* The remarks and details of the author are here neither many, important, nor new. In one form of this complication the blood issues externally, and the labour is not suspended. The placenta, on separation, is found to have an adherent clot attached to it, thus pointing out the spot of disruption. In a case of Mauriceau's, the coagulum was of the size of two fists. In other examples the labour is suspended by the grave effects of the loss of blood. In a third form the flow ceases to be visible; but, nevertheless, is accumulated within the womb, which first does not contract, and then becomes soft, distended, and flaccid, amid all the signs of hæmorrhage already noticed. In all these varieties of flooding, the infant may be affected; and in some, respiration after birth is established but slowly, unless the labour shall have been rapid. In proportion to the advance of gestation is the danger of the effects of flooding. It is rarely fatal before the sixth month. Many, however, remain long enfeebled when the drain has been excessive; the slightest disorders become serious, while some, says M. Gendrin, become prone to uterine congestions, menorrhagia, and to its consequences, frequent abortions.

B. Hæmorrhage, with Abnormal Implantation of the Placenta.—M. Gendrin has the following observation in the very second paragraph of this chapter: "So long as the dilatation of the uterus is confined to its body, that is, from the first to the fourth month of gestation, abnormal implantation of the placenta is unattended by any accident." Now, admitting, with a few authorities, that even from the commencement of pregnancy there is something like a placenta, still the traces are such mere sketches, that the majority of observers have always stated that this organ is non-existent till the third month; hence it is not the want of dilatation of the cervix uteri previous to the fourth month which can be said to prevent flooding. The period at which, in our own experience, we have most often noted its commencement, is after the seventh month. We have never known it as early as M. Gendrin says, viz., after the fourth. In a few instances we can corroborate his statement of the flooding beginning only with labour at the full time. We can also bear our testimony to the fact, that the placenta is not implanted, centre for centre, over the os, in the majority of cases. A lobule, or a portion somewhat larger, edges over the funnel-shaped cervix uteri. The two most remarkable signs which we would notice are the existence of the first gushes, unattended by uterine pain, and their occurrence independent of any assignable mechanical cause. In several instances the flooding has occurred at night when the patient was asleep. The subsequent course of placental hæmorrhage is marked by intermissions of a fortnight, then a week, and ultimately by incessant slight discharge, with occasional slight gushes. The fœtus is always in jeopardy and often is killed; but we have no experience as to the assertion of M. Gendrin, (p. 193,) that the flooding ceases as soon as the child is dead, and that labour takes place after this event: we, however, doubt its justness.

Besides the indication furnished by hæmorrhage occurring at shortening intervals, without apparent cause, during the latter months of gestation, the os uteri does not, previous to real labour-pains, differ much, judging by the touch, from the natural state. It is, perhaps, more "*cushiony*," but the head of the fœtus is not felt; still this negative sign may be the result of some other presentation of the fœtus. With labour-pain, however, the case is cleared up; the dilatation thence induced permits us to feel the spongy placenta, and to become sensible of the cause, which, indeed, was readily to be inferred from the gushes of blood coinciding with uterine contraction, and ceasing in part with uterine relaxation. Fortunately, uterine contraction in these miserable cases is almost invariably slow, while the dilatability of the lower segment of the viscus is usally facile. No one need be reminded of the extreme danger, both to mother and infant, in these cases. But there are others where, although the placenta be abnormally implanted, still the hæmorrhage ceases, and labour proceeds naturally. The fact noted by M. Gendrin we have also observed, viz. that all the signs of "placenta prævia" may have existed during the latter portion of gestation, and yet the labour terminate quite naturally. A portion of the placenta has become detached, and a reparative process has closed up the open-mouthed vessels so as to preclude further bleeding: such is M. Gendrin's explanation in the cases to which we allude. We have found the cessation of the flux to have been dependent, during labour, on pressure of the child's head on the flap of placenta. Wigand (Geburt. des Menschen) has given us six or eight examples of genuine placenta presentations treated by plugging, in every one of which the labour was effected naturally, and without any the least dangerous hæmorrhage.

The following extract is a recapitulation of the appearances produced by hæmorrhage on the ovum:

"1. If the hæmorrhage be slight, one or more deposits will have taken place; the placenta, preserving its natural texture and development around them.

"2. If the hæmorrhage be so considerable as for the blood to have become infiltrated into its whole substance, disorganizing it, this viscus and the fœtus are converted into a foreign body. The latter being killed becomes atrophied, dry, and diminished in size, and suspended in a mass of stratified blood. Sometimes the internal flux continues slowly, in which case the placentary mass enlarges, by addition of fluid, and ultimately is expelled in the form of a spongy body, penetrated and surrounded by a great quantity of liquid blood.

"3. If the hæmorrhage be rapid, coagula surround the ovum, and sometimes lacerate the placenta and membranes, and in a short time the mass is expelled." (p. 210.)

The quantity of blood which may be poured out on the centre of the placenta without detaching its edges from the uterus, we have seen, in the case of Albinus, to have been sufficient to cause death. M. Gendrin quotes another remarkable case of the same kind.

"A woman, thirty-six years of age, the mother of many children, and in her eighth month of pregnancy, had a violent cough and fever. Being seized with labour she sent for the midwife, who, after twelve hours of travail, saw her patient fall into the most alarming state. M. Delaforterie was sent for, but arrived only after her death. He instantly performed the Cæsarian section with every requisite precaution; and on opening the fundus uteri witnessed the escape of a gush of blood, which he estimated at three 'chopines' at least. This fluid left a large pouch between the placenta and uterus, into which M. Delaforterie, on introducing his hand, was satisfied that the placenta had preserved its natural marginal adhesions with the womb. The fundus of the latter organ exhibited no trace of rupture. There was no blood in the vagina; the uterine orifice was little dilated. The child, though extracted alive, lived only a few seconds." (p. 211.)

The extent, however, of this extravasation, must be considered as remarkable as its mode of inclusion. Usually, says M. Gendrin, the clots imbedded in the placenta are small, seldom exceeding in bulk a pullet's egg; oftener they are smaller, and interspersed over the placental surface. They are of a yellowish-gray colour, solid, and, as it were, fleshy; they are, he adds, very common, and would have been oftener noticed were not the examination of the placenta habitually neglected. Their smallness accounts for the safety of the mother, while their presence will explain the debility of the fœtus in many instances, and the atrophied state of the organ, at once impeded in function and restrained in bulk. We would strongly recommend these observations to the reader. Nine-tenths of the abortions are produced by the effects of a disordered utero-placental circulation. We believe, however, that the yellowish-gray masses, described by Gendrin, are, in some instances at least, a disease of the placenta itself, independent of effusion. Such placentæ we have repeatedly examined, and found all those changes going on which are visible in the conversion of cellular membrane into bone: in one part, perhaps, a little thickening, in another a yellowish cartilaginous texture; in a third spiculæ, which feel like bony texture. We need not add that in such examples the after-birth has been adherent, and the child ill-nourished.

In these effusions of blood into the placenta, M. Gendrin remarks that the liquor amnii is usually reddish: and the infant what is termed blighted; and though retained in utero, its arrestation or death is usually coincident with the first symptom of hæmorrhage. These observations tally with our own experience.

With regard to the lesions observable in the placenta where that organ has been implanted on the os uteri, M. Gendrin has dissected, since the year 1824, forty-two such examples. The following observations merit great attention; we must, however, abridge them:

"If the placenta is implanted only in a small extent over the os uteri, the portion so engaged is as friable as the spleen, but looks like the lungs when thrust by pleuritic effusion against the vertebral column. The spongy tissue is obliterated and rendered homogeneous by blood incorporated with it. This, the simplest alteration, is observed only when the hæmorrhage just precedes or even accompanies the expulsion.. In cases where the flooding has occurred at distant intervals, there is a larger segment of placenta implanted, and the alterations are various in three points, viz., 1, the centre; 2, the intermediate zone; and 3, the outer margin of the organ. The central portion is condensed, homogeneous, granular, grayish-yellow in colour, readily friable, and intersected by white filaments, which extend in ramifications to the uterine surface, but are lost in the yellow layer; which does not present, throughout its thickness, a single point of redness. In the midst of the homogeneous tissue small reddish-black clots are seen, usually in great numbers, not separable from, but confounded with its substance. These small clots ordinarily penetrate to the chorion. The surface of the placenta, in these altered portions, presents white spots of a few lines diameter, having the aspect of those tuberculous deposits found on the peritoneum.

The intermediate zone of placenta is red, with coagulated blood infiltrated into, and incorporated with its texture. It is much softer and more friable than in the healthy organ. Its homogeneous appearance is broken up by cysts of blood of varying depths. The exterior zone has the same aspect as that described as exhibited in partial implantation of the placenta." (p. 217.)

From this description M. Gendrin concludes that the lesions mark the epochs of hæmorrhage, the central zone being the result of the earliest. The other lesions depend on ruptures produced either by the uterine expulsive efforts, or those incident to extraction by the accoucheur.

M. Gendrin ascribes the hæmorrhage "unavoidable," as it is termed, not to separation but to rupture of the placenta from the uterus. The alterations into the yellowish-gray matter just noticed is a process of reparation by means of which, as already observed, the flooding of the earlier months is stayed during the latter; and the labour, though a placental one, is not necessarily hæmorrhagic.

*Causes of utero-placental hæmorrhage.* Pregnancy, by establishing a natural congestion in the uterus, must of course be regarded as the predisposing cause of the flooding which often attends it. Menstruation, too, or the habit thence induced, is another; and we have before assented to M. Gendrin's remark, that the floodings of gestation coincide, for the most part, with what would have been in the unimpregnated state a menstrual period. Besides these, there are certain temperaments which predispose to uterine bleeding: 1. The sanguine: marked by a powerful circulating apparatus. 2. The lymphatic: characterized by a languid transmission of the blood by means of a feeble heart and arterial system, by a great development, on the other hand, of the capillaries, and by a large though feeble frame. 3. The nervous: fragile in form, pale in aspect, but full of pains which flit from organ to organ: these women, for the most part, are subject to irregular uterine gushes, from extreme uterine irritability.

Mechanical causes acting on the uterus, it is well known, will produce flooding; but there must be a predisposition to it, judging from the numerous examples in which the concussion does not end in discharge. Nevertheless, Smellie has noted this result as succeeding violent vomiting; Van Swieten, sneezing; and others have remarked a similar effect as resulting from the spasms of an hysteric fit.

Moral impressions of a violent or intense kind, whether exciting or depressing, doubtless must be accounted as frequent and effective occasional causes. (*To be continued.*)

---

*Remarks on Collapse occurring during the treatment of Acute Pneumonic Diseases.* By William Kerr, Surgeon, Corresponding Member of the Medical and Physical Society of Calcutta.—The object of the following remarks is to call the attention of the medical public to a dangerous train of symptoms immediately succeeding, in some instances, the inflammatory stage of pneumonia and pleurisy. In those to which I allude, a state of sinking suddenly and unexpectedly occurs within a day or two after the removal of the pain, when the patient is much easier, and apparently about to be, in a short time, restored to health. The cases which I shall relate, were not originally intended for publication, and probably would never have been printed, had I not found, from conversation with several gentlemen of considerable celebrity in the profession, that they were ignorant, not only of the treatment, but of the occurrence of the above dangerous attendant upon acute pneumonic complaints. With this apology for cases which do not possess the copiousness of detail I could have wished, I proceed to relate them, hoping that they may at least serve as guides till farther and more minute observations are made.

I. A message was sent to me one evening in the end of March, 1836, requesting a visit to a very respectable and temperate-living weaver, fifty years of age; but, owing to other engagements, I was unable to see him till next forenoon, when I found him complaining of severe pain in the right side of the chest, preventing him from lying in the horizontal position in bed, and aggravated by inspiration and coughing. He said that he had been very ill all night, and wearied very much to see me. The pain had come on some days previously in consequence of cold, but had not acquired much severity till twenty-four hours before my visit, about which time cough commenced. I immediately opened a vein, and abstracted sixteen ounces of blood with such great and immediate relief, that I flattered myself that the disease was subdued. I gave a dose of calomel, which I happened to have with me, and paid attention to secure a comfortable warmth of body, a flannel jacket being put on, and a small jar filled with hot water laid to the feet.

Next day, the pain having returned during the night, I again bled him to the extent of sixteen ounces, with immediate relief. However, in a few hours, there was a return of pain, but in a very mitigated degree, for which a blister was applied. Next day he was better, though not free from pain; next again, he was so well that, on entering the house, I found him its only inmate, his wife having gone out for a short time, thinking that he now required little attention. Previously to this day, his breathing during sleep was always oppressed, but it had now become easy. During the whole illness, however, he had little sleep. On the succeeding day, the fifth of my attendance, to my astonishment, I found him much worse, his pulse frequent, and his manner abstracted, like that of a patient in typhus fever, to which disease the symptoms now bore a strong resemblance. In the evening he began to be incoherent. Next afternoon, he was quite insensible, and unconscious of being in existence. Bewildered with symptoms which I did not anticipate or comprehend, I knew not what to do, and in twenty-four hours he died.

2. On the 2d of August, 1836, I was sent for in haste to visit a farmer, of most exemplary and temperate habits, fifty-eight years of age, who had gone that evening to drink tea with another farmer, fully more than a mile from his own house. After tea, while walking in the fields with his friend, he was seized with pain in the left side of the chest, which was so severe that it was necessary to convey him to the nearest house, where I found him awaiting the arrival of a carriage. The pain soon abated very considerably, and on his way home he expressed himself as being much better. On getting to bed care was taken to clothe him in flannel, and to apply bottles or small jars filled with hot water. Notwithstanding these, however, the pain suddenly became violent; it was not accompanied with cough, but it rendered inspiration or the slightest movement of the body very painful, and prevented him from lying on the affected side. I now opened a vein, but the result of last case having made me timid, I abstracted scarcely a soup-plateful of blood, with very little mitigation of the pain. To remove the remainder I gave a dose of opium, which was repeated during the night, but without benefit. His bowels were kept open by purgatives and enemata. Two sinapisms and a blister were applied likewise inefficaciously, and he died about eighty-four hours from the commencement of the illness. About a day or rather more before death the pain somewhat abated, but the breathing became more frequent, and the oppression greater. From the commencement of this period, he expressed a wish to be allowed wine, in which he was indulged to the extent of three or four wine-glassfuls. Incoherence was at no time present. During the whole illness, though slumbering occasionally from the few doses of opium administered, he could not be said to sleep. On the day of his death he was able for the first time to lie on his left side, and imagined himself to be better.

3. On the 17th of June, 1837, I was sent for to visit a farmer, forty-one years of age, of temperate and steady habits, and previously in good health. I found no decided symptom of any complaint. He felt himself unwell, his body generally pained, and his appetite bad. These he attributed to cold caught two days previously, by standing still in the evening air without his coat in a state of perspiration from hard work, talking for a considerable time to a friend. I caused him to take a purgative, to put on a flannel jacket, and to go to bed with a jar filled with hot water at his feet. Next morning a messenger was sent to inform me that severe pain in the lower part of the left side of the chest had come on during the night. I directed one grain of opium to be taken, and a mustard cataplasm to be applied immediately. Two hours afterwards I visited him, and found that he was not in any measure relieved. The pain in the chest was severe, preventing free inspiration, and obliging him to lie on the opposite side, and was attended with cough, by which it was greatly aggravated.

Suspecting that in the last case the inflammation had never been so completely subdued as it should have been, owing to timidity in taking away the requisite quantity of blood, and that, in the first case, I had possibly taken too much, I began to be of opinion, that the fatal sinking which so unexpectedly followed the abstraction of blood might perhaps have been remedied, had stimulants been given freely whenever the change of symptoms appeared. I therefere determined in the present instance to put a stop, if possible, to the inflammation in as short a time, and with as little loss of blood as possible, and to give wine liberally, as soon as any degree of delirium should appear. Accordingly, I opened a vein, and abstracted nearly a soup-plateful of blood. Finding at the end of an hour that the pain was mitigated, but not removed, I re-opened the wound, and allowed more blood to flow till he had lost altogether twenty-four ounces. This abstraction of blood gave great relief, and after having waited for several hours, I left him, satisfied with the result of the treatment. Next day the pain was quite trifling, though still felt, and he was evidently much better, On the succeeding day, the 21st, the pain not being wholly gone, tartar emetic was given in small doses, but it having sickened him, he refused to persevere in its use. A grain of opium was given at bed-time to procure sleep, Indeed, since he began to be indisposed, he had slept ill. In the morning of the 22d, a purgative which he had taken operated six or seven times, and feeling himself, as he imagined, much better, notice was sent, at his request, that I might dispense with visiting him that day.

Aware, however, of the deceitful nature of the disease, I disregarded the message, and on my arrival found him complaining peevishly of the opium having produced a restless night, and uneasy dreams, and some of his expressions were decidedly incoherent. His pulse likewise was frequent. I now learned that since the evening of the 19th, he had talked incoherently when slumbering, and that since the same time, he had manifested a good deal of obstinacy and peevishness. He had had no appetite since the day of my first visit. I ordered a glassful of wine to be given at least every three hours, and gave ten drops of Battley's sedative liquor of opium, which I happened to have with me, to be repeated every six hours till sleep should be procured. After commencing this treatment, I left him for two hours, and on my return, found the frequency of the pulse somewhat lessened. On the 23d, I was informed that sound sleep had not come on till after the third dose of sedative liquor, but that since that time he had slept with little intermission. He relished the wine, and had taken it as directed. This night he slept without opium; next day he was only slightly incoherent. By the 26th, he had some appetite, and swallowed a small quantity of beef-tea; by this time, too, he had little relish for wine, but the symptoms being greatly improved, it was not considered necessary to urge him to take much, and not many days afterwards, he ceased to take any. On the 1st July he was so much better, that I discontinued my attendance. For a long time after he began to go about, he had rather a feeble appearance, and did not regain his original strength till he had reccurse to several glassfuls of wine daily for some weeks.

4 and 5. Besides those cases which I have now sketched, I have heard of a few of the same kind, in which the patients after having been bled with relief, sunk suddenly, and not long ago I met with two instances in which recovery took place in consequence of stimulants being freely given as soon as symptoms of sinking occurred, after the removal of the inflammatory symptoms by blood-letting. One was a middle-aged servant maid, in whom the primary disease was pleurisy, which was followed by a large abscess or empyema in the left cavity of the pleura, which ultimately burst into the bronchiæ. The other was an elderly gentleman whom I saw in consultation, and to whom wine was given in quantities of a wine-glassful every three hours, but not before the raving had terminated in very considerable insensibility. Wine, together with small draughts to procure sleep, and a well-aired room, had the desired effect, and a perfect recovery ensued.

I do not suppose that the sinking now described is confined to pneumonic affections, but possibly may follow other inflammatory diseases if the severity of the attack renders necessary the abstraction of a greater quantity of blood than the constitution can support. Several years ago I had charge of a case of enteritis, in which, owing to two severe attacks occurring in less than a week, I deemed the loss of a considerable quantity of blood requisite. Typhoid symptoms soon followed, and I not being at that time aware of the appropriate treatment, the patient died after having been incoherent, and latterly quite insensible.

16*th May* 1839.

Since the preceding remarks were prepared for press, I have met with another case in which, soon after blood-letting for acute pneumonic symptoms, sinking occurred, which was checked, and recovery produced by the prompt and liberal administration of wine.

6. In the end of May, a gentleman, 24 years of age, was seized with pain the left side of the chest, which at first seemed to be of a spasmodic nature, and diminished greatly under the use of opium, and the application of warmth. Opium was required for three days, but on the fourth the pain was so much easier that he did not take any. On the fifth, he felt some pain in the left side of the chest, impeding coughing; at night this suddenly increased to such an alarming degree, that he durst hardly cough, and was obliged to lie on his back, having his head and shoulders considerably elevated; still the expectoration had no reddish tinge. Pulse 120.

This patient had for several years been confined with phthisical symptoms arising from disease in the right lung, and he had not till within a year or two recovered any measure of health. On this account scarcely more than ten ounces of blood were abstracted. The pain was immediately relieved so much, that he could lie in the recumbent position. The blood on cooling exhibited a buffy coat. Notwithstanding this relief he was through the night very restless and uneasy. In the course of the following day, opium was given to allay these symptoms, but with only imperfect success. Next morning, thirty-six hours after the blood-letting, learning that he did not enjoy above five or ten minutes sleep at once, and that he was incoherent for some time after he awoke, symptoms which occurred in the early stage of sinking in the other patients, wine was prescribed in quantities of a glassful every three hours. He was still not altogether free from pain in the chest, and the cough would have been very troublesome had he not taken opium. Pulse 126; there was considerale thirst, but the tongue was not dry. In the course of the day, after commencing wine, he was observed to sleep longer, and to be less incoherent on awaking. Next morning the pulse was 116; he relished the wine, and felt stronger. On the succeeding day the pulse was 104, and the incoherence gone; the wine was still relished, and taken in the same quantities. At the end of a week he was so much better, that the doses of wine were greatly diminished.

In the summer he went to the sea shore, where he became strong enough to walk out of doors daily. In the beginning of winter, cough became more troublesome, and he was under the necessity of confining himself to bed. Nearly three months ago, a small abscess formed over the original site of the pain in the right side, and was opened, when purulent matter was discharged, of the same appearance as that expectorated. This new outlet has been of much utility, for since it took place, the quantity of purulent matter secreted has progressively diminished, and is now so much lessened that the wound is nearly closed.—*Edinburgh Med. and Surg. Journal.* April, 1840.

*Paisley*, 9th January, 1840.

